# Characterization of the PaHAK Gene and Its Expression During the In Vitro Seed Germination of Two Botanical Avocado Varieties Under Saline Stress

**DOI:** 10.3390/life14121680

**Published:** 2024-12-18

**Authors:** Essoh Aimé Cesaire Elekou, Luis María Suárez-Rodríguez, Mariela Gómez-Romero, Jannette Sofia Bayuelo-Jiménez, Andrés Belver, Juan Carlos Díaz-Pérez, Rodolfo López-Gómez

**Affiliations:** 1 Instituto de Investigaciones Químico-Biológicas, Universidad Michoacana de San Nicolas de Hidalgo, Morelia 58000, Michoacán, Mexico; 1651155h@umich.mx (E.A.C.E.); lsuarez@umich.mx (L.M.S.-R.); 2 Facultad de Biología, Universidad Michoacana de San Nicolás de Hidalgo, Morelia 58000, Michoacán, Mexico; mariela.gomez@umich.mx; 3 Instituto de Investigaciones Agrícolas y Forestales, Universidad Michoacana de San Nicolás de Hidalgo, Morelia 58000, Michoacán, Mexico; bayuelo@umich.mx; 4 Departamento de Estrés, Desarrollo y Señalización en Plantas, Estación Experimental del Zaidin, Consejo Superior de Investigaciones Científicas (C.S.I.C.), 18008 Granada, Spain; andres.belver@eez.csic.es; 5 Department of Horticulture, The University of Georgia, Tifton, GA 30602, USA; jcdiaz@uga.edu

**Keywords:** HAK transporter, *Persea americana* varieties, saline stress, seed germination

## Abstract

Soil salinity is one of the main challenges that modern agriculture faces. Avocado, which is classified as a glycophyte, is very sensitive to salt stress. There are botanical varieties of avocado that differ in their salt tolerance. This study investigated how salt stress affects the in vitro germination of two avocado botanical varieties americana (West Indian breed) and drymifolia (Mexican native) with different salt tolerances. This study also assessed the potential role of the avocado *PaHAK2* high-affinity K^+^ transporter HAK/KUP/KT in response to saline stress during germination. Salinity (60 mM NaCl) delayed the germination speed of the drymifolia variety relative to the americana variety. A computational 3D inference protein model of the *PaHAK2* protein showed 10 highly conserved transmembrane domains. During the imbibition period, there was a differential increase in the expression of the *PaHAK2* gene at 60 mM NaCl in both varieties, which suggests the presence of osmotic adjustment and regulation. The enhanced expression of *PaHAK2* in the americana variety suggests an adaptive advantage to salinity. We conclude that *PaHAK2* participates in the response of avocado to salt stress during seed germination.

## 1. Introduction

Salinity poses a significant challenge to agriculture worldwide, impacting plant growth and development. The detrimental effects of salinity on crops have been extensively documented [1,2]. High levels of soluble salts in soil and irrigation water make salinity the second-largest limiting factor for plant growth and yield, with crops such as avocado being particularly susceptible [3]. Three ecological botanical varieties of avocado have been reported: *Persea americana* var. americana (West Indian), *Persea americana* var. drymifolia (Mexican native), and *Persea americana* var. guatemalensis (Guatemalan). Avocado’s sensitivity to salt stress is well recognized, with differences observed between botanical varieties. The Mexican variety is considered to be the most sensitive to salinity, the Guatemalan variety demonstrates intermediate salt tolerance, and the West Indian variety is the most tolerant to saline conditions. The native Mexican variety (drymifolia) has been used as the main rootstock for the Hass avocado cultivar globally, and the americana variety has been used as an alternative in high-salinity areas [4,5,6,7,8]. The rootstock affects the fruit weight and health of ‘Hass’ avocado trees exposed to saline water [9]. Consequently, avocado’s response to salt stress likely changes depending on the botanical variety and even the phenological stage of growth [10,11]. Salt-induced osmotic stress affects various physiological processes, including germination, imbibition speed, and germination velocity [12,13]. Research on the effect of salinity on avocado germination has consistently shown that var. americana has higher germination rates than var. drymifolia and cv. Hass [4,10]. Similar to the germination stage, an adverse effect of salinity was also reported on avocado tree growth, mineral nutrition, and productivity [9,14,15]. These contrasting germination responses may be due to genetic variation or differential gene expression [16]. Advancements in genomic and transcriptomic studies in avocados have facilitated the identification of genes with potential for biotechnological improvement [17,18,19,20,21,22]. However, the genetic basis of the salt tolerance of avocado botanical varieties is unknown. Potassium (K^+^), as the most abundant inorganic ion in plant cells, plays a crucial role in metabolic, physiological, and developmental processes [23,24]. High external Na^+^ concentrations competitively inhibit K^+^ uptake systems and stimulate K^+^ efflux, leading to insufficient cellular K^+^ concentrations for enzymatic reactions and osmotic adjustment [25,26]. Therefore, maintaining a cytosolic balance of the Na^+^/K^+^ ratio is a key salt-tolerance mechanism in plants. Under saline stress, K^+^ absorption and distribution occur through specialized K^+^ channels and transporters [27], among which, the HAK/KUP/KT transporter and ion channel proteins play a crucial role in K^+^ uptake and transport. Most HAK genes have been described principally for herbaceous plants and a few have been described for woody plants [28]. In the native Mexican avocado (*Persea americana* var. drymifolia), transcriptome analysis revealed the abundant expression of a gene encoding a K^+^ transporter/high-affinity K^+^ transporter/K^+^ uptake protein (KT/HAK/KUP) during the initial phase of seed development [20]. However, the function of this K^+^ transport protein during germination in avocado, as well as its potential role in these processes under saline conditions, remains unknown. Studies in rice have identified quantitative trait loci (QTLs) associated with salt tolerance, some of which encode K^+^ transporters [16,29,30,31]. For instance, rice QTL qSE3, which encodes the K^+^ transporter/high-affinity K^+^ transporter/K^+^ uptake protein (KT/HAK/KUP) *OsHAK21*, exhibits enhanced expression under saline stress during rice seed germination and seedling establishment [16]. Variation in the coding sequence of *SlHAK20*, which transports Na^+^ and K^+^ and regulates Na^+^ and K^+^ homeostasis under salt stress conditions, was found to be the causative variant associated with the Na^+^/K^+^ ratio and conferred salt tolerance in tomato [32]. This study aimed to investigate how salt stress affects the in vitro germination of the avocado botanical varieties *Persea americana* var. americana (West Indian breed) and *Persea americana* var. drymifolia (Mexican native), which differ in their degrees of salt tolerance. The characterization of avocado *PaHAK2*, a high-affinity K^+^ transporter HAK/KUP/KT gene, and its expression in response to saline stress was assessed via real-time quantitative PCR during the germination of avocado seeds.

## 2. Materials and Methods

### 2.1. Plant Material and Experimental Design

Ripe avocado fruits of the varieties *P. americana* var. drymifolia (Mexican native) and *P. americana* var. americana (West Indian breed) were acquired from the local markets of Morelia, Michoacán, and Mérida, Yucatán, Mexico. The fruit seeds were extracted and germinated following a completely randomized design comprising 3 repetitions of 20 seeds per treatment (4 treatments) for a total of 240 seeds per variety. 

### 2.2. In Vitro Germination in the Presence of NaCl

Avocado seeds of the drymifolia and americana varieties were subjected to an in vitro germination protocol [33]. Briefly, the seeds were disinfected via immersion in a 10% (*v*/*v*) solution of commercial sodium hypochlorite (Cloralex^®^, Monterrey, Mexico 6.0% *w*/*w*.) for 10 min. The seeds were then washed three times with sterile water, followed by immersion in 70% (*v*/*v*) ethanol for 15 min. Finally, the excess alcohol was removed, and the seeds’ surfaces were flamed in a laminar flow hood. Cuts were made in the cotyledons, leaving 1.5 cm sections per side surrounding the embryo. The explants were plated in MS basal media [34] supplemented with 0.05 mg/L of 6-benzyl amino purine (BAP) (Sigma Cat. No. B3408, St. Louis, MA, USA), 30 g/L of sucrose (Bioxon, Cat. No. 217000, Franklin Lakes, NJ, USA), 8 g/L of agar plant (Phyto Technology Lab, Lenexa, KS, USA. Cat. No. A111, USA) and NaCl treatments (0, 15, 30 and 60 mM). The pH of the culture media was adjusted to 5.7–5.8. The media were autoclaved at 120 °C for 15 min. The seeds were maintained in a growth chamber at 25 ± 1 °C with a 16 h light/8 h dark photoperiod. Root emergence was used to determine the germination percentage; germination is evidenced by an increase in separation between the remnants of the cotyledons that surround the embryonic axis. Daily observations of the seeds were carried out for 15 days. To determine the effect of the NaCl concentration on germination, the percentages of accumulated germination and the time necessary to reach 25% (T25), 50% (T50) and 75% (T75) germination were assessed. The percentage of germinated seedlings and the germination speed (GS) were also recorded [35]. For molecular studies, material was harvested in groups of three seeds at 0, 3, 6, 9, 12 and 24 h (imbibition time) and 3, 5 and 10 days of germination time.

### 2.3. Analysis of PaHAK2 Gene Expression

Specific oligonucleotides were designed with the help of the Primer3 webtool v.4.1.0 (https://primer3.ut.ee, accessed on 16 December 2024), generating the primers *PaHAK2* (NCBI: PP851826) Fw 5′-GCATTGCAGAGGTTGGTGTG-3′ and *PaHAK2* Rv 5′-CCGCCCTCCTTGATCTTT GT-3′ for the target gene and the primers *PaSUMO* Fw 5′-GATAAGAAGCCCACGGATCA-3′ and *PaSUMO* Rv 5′-GACGGCCATCGAATAAGAAC-3′ for the *PaSUMO* (Small Ubiquitin-like Modifier; NCBI:PP851827, https://www.ncbi.nlm.nih.gov/nuccore/PP851827.1/, accessed on: 4 June 2024) housekeeping gene. RNA extraction was conducted using seeds and seedlings harvested at different experimental times, following the modified method [36]. We utilized 1 µg of total RNA for cDNA synthesis using a RevertAid H minus First Strand cDNA Synthesis kit (Thermo Scientific, Waltham, MA, USA, Cat. No. EP0451, USA), following the manufacturer’s instructions. Gene expression levels were determined via qPCR, using a standard curve and relative quantification (ΔCT) for each gene. Relative expression was calculated from the difference in the threshold cycle between the gene studied and the DNA amplified by specific primers, using *PaSUMO* as a housekeeping gene. Each amplification reaction consisted of a 10 μL qPCR (200 ng/rxn cDNA 1:5 dilutions; 1 μM of each oligo; 7 μL of Máxima SYBR green/ROX qPCR kit MasterMix (Thermo Scientific, Waltham, MA, USA, Cat. No. K0222, USA) using Applied Biosystems StepOne equipment (Thermo Scientific, Waltham, MA, USA), with the following amplification program: 94 °C for 10 min and 40 cycles (94 °C, 15 s; 60 °C, 30 s; and 72 °C, 30 s); each sample had three technical replicates. 

### 2.4. Bioinformatic Analysis of the PaHAK2 Gene

A bioinformatics analysis of the homology and phylogeny of the HAK-type potassium transporter of *P. americana* var. drymifolia was conducted to compare it with those of different sequenced plant genomes (*Arabidopsis thaliana*, *Amborella trichopoda*, *Beta vulgaris, Capsicum annum*, *Citrus clementina, Cucumis melo*, *Glycine max, Medicago truncatula*, *Musa acuminata*, *Oryza sativa*, *Phaseolus vulgaris*, *Prunus persica*, *Solanum lycopersicum*, *Solanum tuberosum*, *Sorghum bicolor, Triticum aestivum*, *Vitis vinifera*, and *Zea mays*) extracted from the EMBL-EBI database using the Ensembl Genomes Plants BLAST sequence similarity search webtool (http://plants.ensembl.org/Multi/Tools/Blast, accessed on 16 December 2024). Analysis of the motifs was performed using Geneious Prime v2024.0.4 software (https://www.geneious.com/updates, accessed on 16 December 2024), Interpro (https://www.ebi.ac.uk/interpro/, accessed on 16 December 2024) and AlphaFold (https://alphafold.ebi.ac.uk/, accessed on 16 December 2024), using PDB-based SMTL accessions as a search model (https://swissmodel.expasy.org/, accessed on 16 December 2024). A phylogenetic model tree was constructed based on substitution and evolutionary distances using the Jukes–Cantor genetic model with the UPGMA (Unweighted Pair-Group Method with Arithmetic mean) and a bootstrap resampling method with a value of 500. The 3D protein structure was modeled via homology using the SWISS-MODEL web-based tool, and the identification of possible structural templates was enabled via the BLAST-based alignment of the query sequence with the structures used as templates. Finally, modeling and quality assessment were performed to select the most suitable structure.

### 2.5. Statistical Analysis

Seed germination was analyzed through a generalized linear model (GLM) for data with a binomial distribution. The data were previously verified using the Shapiro–Wilk and Levene tests to confirm that the variables met the assumptions of normality and homoscedasticity. The treatments were compared by means of an analysis of variance (two-way ANOVA) accompanied by Tukey’s test (*p* ≤ 0.05). Statistical analyses were carried out using the Statistica v.10.0 program for Windows [37].

## 3. Results

### 3.1. In Vitro Avocado Seed Germination Under Salt Stress

**Figure 1:**
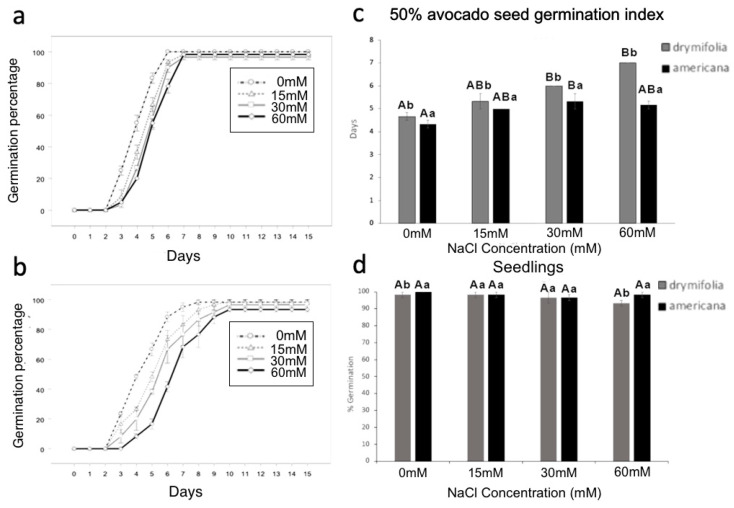
Seed germination and seedling establishment under salinity conditions (0, 15, 30, and 60 mM) of *P. americana* var. americana (**a**) and *P. americana* var. drymifolia (**b**). Time for 50% of the germination percentage (**c**). Germination percentage for both varieties (**d**). Different letters atop the bars indicate significant differences (Tukey test *p* ≤ 0.05). Capital letters indicate intravariety differences; lowercase letters express intervariety differences in NaCl.

There was a significant difference (*p* = 0.000031) in germination speed between the NaCl treatments and between varieties (Figure 1, Tables S1 and S2). In both varieties, the control (0 mM NaCl) had a relatively shorter latency time with respect to the NaCl concentration. Both varieties showed differences between salinity treatments with respect to the time required to reach 50% germination (Figure 1a–c), germination speed, and the time to reach the maximum number of seedlings (Tables S1 and S2, Figure 1d). For both varieties, the germination speed decreased with increased NaCl concentration. This effect was more marked in the drymifolia variety. The 60 mM NaCl concentration resulted in the lowest seed germination percentage in both varieties; 50% of the germination time for the americana variety was reached at 5 days, and drymifolia reached this value at 7 days (Figure 1c). The germination percentage for both varieties was 93–100% at 15 days. The americana variety was less affected, suggesting it had increased tolerance to salinity. There were no significant differences in the germination percentage (range: 96.67% to 100%) between varieties and NaCl concentrations at 15 days (Figure 1d).

### 3.2. Phylogenetic Analysis of the Avocado HAK Gene

**Figure 2:**
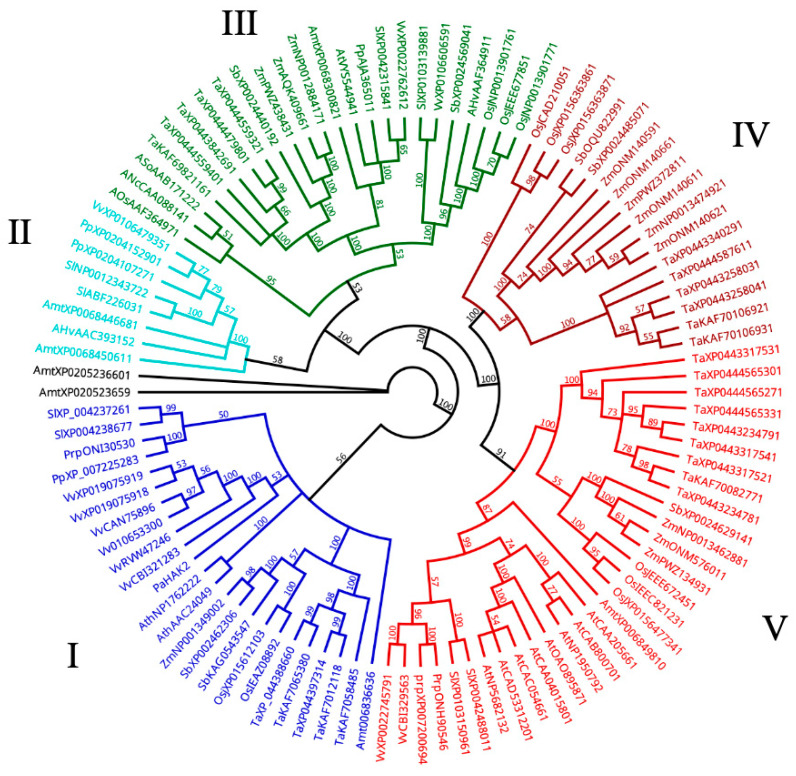
Avocado *PaHAK2* gene phylogenetic tree. UPGMA phylogenetic tree (Jukes–Cantor model) of HAK proteins from *Arabidopsis thaliana* (At), *Amborella trichopoda* (Amt), *Beta vulgaris* (Bv), *Capsicum annum* (Ca), *Citrus clementina* (Cc), *Cucumis melo* (Cm), *Glycine max* (Gm), *Medicago truncatula* (Mt), *Musa acuminata* (Ma), *Oryza sativa* (Os), *Phaseolus vulgaris* (Pv), *Prunus persica* (Pp), *Solanum lycopersicum* (Sl), *Solanum tuberosum* (St), *Sorghum bicolor* (Sb), *Triticum aestivum* (Ta), *Vitis vinifera* (Vv), and *Zea mays* (Zm) were obtained from the EBI-EMBL database. The construction was carried out with 106 sequences. The different branch colors represent different clusters. The numbers on each branch show the bootstrap values calculated for 500 replicates.

**Figure 3:**
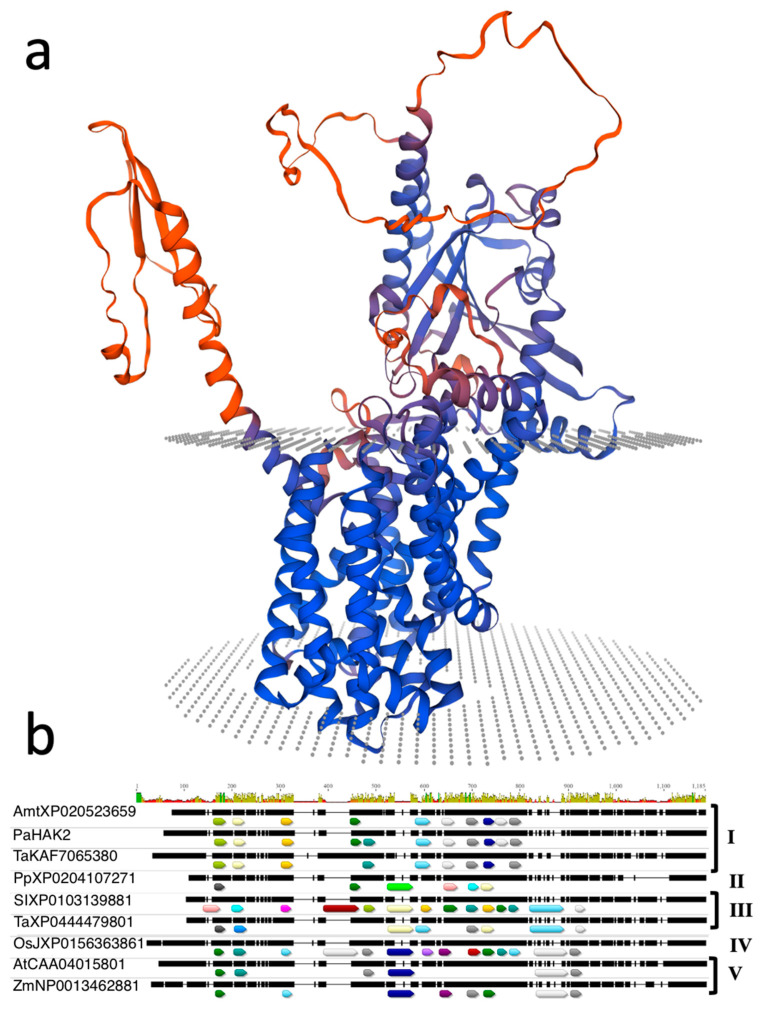
(**a**) Molecular modeling of the *PaHAK2* protein in *Persea americana* var. drymifolia. The 3D model was constructed using the SWISS-MODEL web-based tool based on the potassium transporter AlphaFold structure of *Kingdonia uniflora* (A0A7J7NQB0.1.A) that shares 79% sequence similarity. (**b**) The most representative sketch map of conserved motif distribution in high-potassium transporter proteins. The protein length scale is presented at the top. Each motif is represented by a colored box on the bottom. *Amborella trichopoda* (XP_020523659.1), *Persea americana* var. drymifolia *PaHAK2* (PP851826.1), *Triticum aestivum* (KAF7065380.1), *Prunus persica* (XP_020410727.1), *Solanum lycopersicum* (XP_010313988.1), *Triticum aestivum* (XP_044447980.1), *Oryza sativa* Japonica Group (XP_015636386.1), *Arabidopsis thaliana* (CAA0401580.1), and *Zea mays* (NP_001346288.1) are considered representative sequences of each phylogenetic branch classification; similar colors represent similar motif composition.

In a transcriptome study of immature avocado seeds, we found that the *PaHAK2* gene was abundantly expressed [20]. The avocado *PaHAK2* gene belongs to cluster I of K^+^ transporters with different transport activities. The protein sequence of avocado *PaHAK2* was used to find HAK/KUP/KT transporter orthologs in selected genomes. A total of 106 sequences obtained were used to construct a phylogenetic model tree. A total of five clusters (I–V) were obtained (Figure 2). The HAK/KUP/KTs clades are consistent with previous reports [28,38]. In the case of *PaHAK2*, 10 highly conserved motifs were found throughout cluster I. The differences in size and the presence of these conserved motifs helped resolve the topology of the phylogenetic tree. The 3D structure inferred for *PaHAK2* (Figure 3b) using the SWISS-MODEL web-based tool (Swiss Institute of Bioinformatics Biozentrum, Basel, Switzerland) used the sequence A0A7J7NQB0.1.A, which belongs to the *Kingdonia uniflora* K^+^ transporter, as a template. Interestingly, *Medicago truncatula* is the only species in the cluster that has the motif (GVVYGDLGTSPLY) absent, presenting only 10 conserved motifs consistent with what was previously reported [32].

### 3.3. PaHAK2 Expression During Seed Germination Under Salt Stress

**Figure 4:**
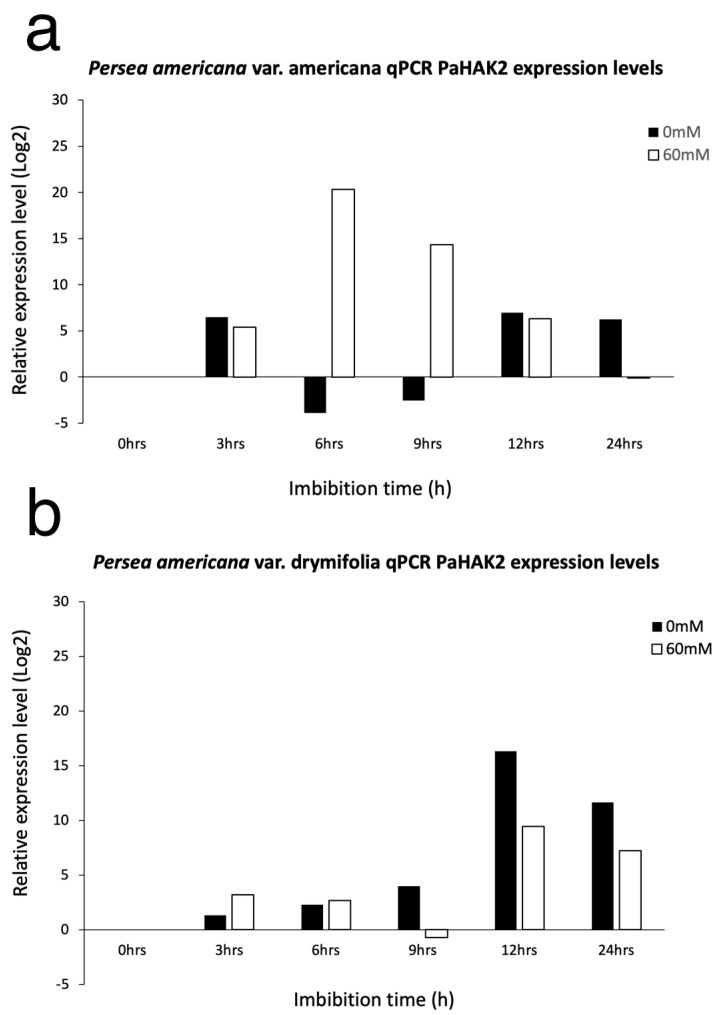
qPCR *PaHAK2* gene expression differences between the americana (**a**) and drymifolia (**b**) varieties during seed imbibition under saline stress (60 mM NaCl). The relative expression levels are represented by the fold change normalized to that of the *PaSUMO* control gene expression.

**Figure 5:**
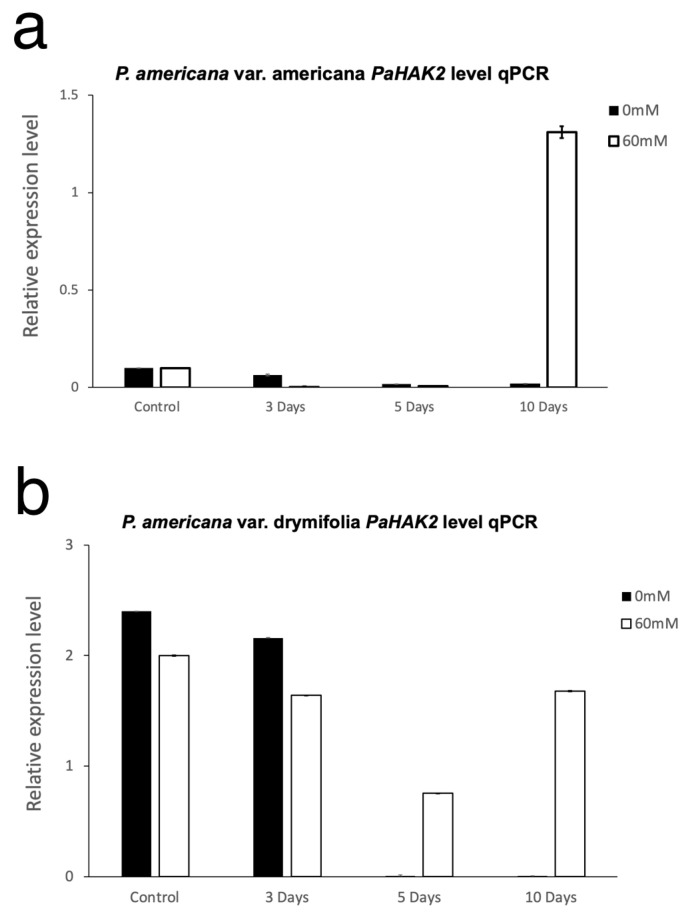
qPCR interspecific *PaHAK2* expression differences between the americana and drymifola avocado varieties during seed germination under saline stress (60 mM NaCl). Americana variety (**a**), drymifolia variety (**b**). An increase in expression was observed 10 days after germination. Gene expression was normalized to the *PaSUMO* control gene. Data are presented as means ± standard deviation (*n* = 3).

We performed qPCR analysis to determine whether the expression levels of *PaHAK2* were regulated by high salinity during seed germination for the two avocado varieties (Figure 4 and Figure 5). During the imbibition period in the americana variety at 0 mM NaCl, there was a decrease in the expression of the *PaHAK2* gene at 6 and 9 h and the expression increased at 12 and 24 h. At 60 mM NaCl, gene expression increased at 9 h, followed by a decrease until a minimum was reached at 24 h. The increase in expression was notably greater at 60 mM NaCl compared with 0 mM NaCl (Figure 4a). However, in the drymifolia variety, there was a gradual increase from 3 h of imbibition until a maximum was reached at 12 h and there was a decrease at 24 h at 0 mM NaCl concentration. At 60 mM NaCl, *PaHAK2* gene expression gradually decreased to a minimum at 9 h, increasing again at 12 h and decreasing again at 24 h (Figure 4b). Comparing the expression levels of the two varieties, there is a stronger increase in the americana variety under salt stress conditions than in the drymifolia variety. At 0 mM NaCl, the expression of *PaHAK2* was minimal in the americana variety during the first 10 days of germination (Figure 5a). However, at 60 mM concentration of NaCl, we observed an increase in expression at 10 days (Figure 5a). In the drymifolia variety, we observed that basal *PaHAK2* gene expression in the control (0 mM NaCl) gradually decreased as germination progressed (Figure 5b). At 60 mM concentration of NaCl, gene expression decreased until the fifth day. Gene expression was induced on the tenth day in both varieties (Figure 5a,b).

## 4. Discussion

Saline stress negatively influences crop production worldwide because most cultivated plants are salt-sensitive “glycophytes”. Na^+^ and Cl^-^ ions are common leading causes of salinity due to their high concentrations in soil. Salt stress affects seed germination and seedling establishment [39]. Salinity affects seed germination through osmotic stress, ion-specific effects, and oxidative stress [40]. Avocado’s sensitivity to salt stress is well recognized, with differences observed between botanical varieties due to genetic variability; some individuals can tolerate salinity better than others. Among the three ecological botanical avocado varieties, var. americana is known to be tolerant to salinity, while var. drymifolia (Mexican native) is the most sensitive, and even low NaCl concentrations affect its development [7,8,41]. Our results show that NaCl differentially influences the germination of the two varieties of *P. americana*. Tolerance to salinity is significant for survival, and it is reflected in the germination speed (GS) and germination index as salt concentrations increase. A noticeable increase in germination time can be observed with higher concentrations of NaCl (Figure 1a,b). These results are consistent with reports in beans, tomato, Arabidopsis, rice, and sorghum, where an increase in germination time was observed with increased NaCl concentration [13,16,42,43,44]. In short, the osmotic effect caused by high NaCl concentrations affects germination by delaying seed metabolic activation, which favors root emergence and subsequent seedling development [45,46]. Although the germination speed was reduced to 60 mM, there was no difference in the final germination percentage between varieties. This could be because responses to NaCl may vary depending on genotype [47]. The reduced impact of salinity in var. americana compared with var. drymifolia agrees with other reports [4,5,6,7,8,9,10]. In plant cells, K^+^ is the most abundant inorganic ion. Several K^+^ transporters, such as KT/KUP/HAK, have been found to be essential for salinity tolerance in plants [16,32]. Most HAK genes have been described principally for herbaceous plants, and a few have been described for woody plants [28]. The HAK/KUP/KTs clades are consistent with previous reports [28,38]. The avocado *PaHAK2* protein is present in cluster I, close to the proteins of *Vitis vinifera* and *Amborella trichopoda*. *PaHAK2* in the basal clade denotes the importance of K^+^, which is used by cells as the principal cation for essential functions such as the maintenance of electroneutrality and osmotic balance. Therefore, it is interesting to find that *PaHAK2* is found in the most basal positions of the phylogenetic tree generated in this study, denoting the orthology of these transporters present in avocado and coincidentally also corresponding to the basal phylogenetic distance of angiosperm families found on the Angiosperm Phylogeny Website (APG IV). The clusters comprise proteins from multiple species, suggesting that the protein families of the K^+^ transporters represented by the calculated clusters are evolutionarily conserved. We analyzed conserved transmembrane protein motifs of the similar protein sequences found in the genomes used, which helped us identify typical topologies for each calculated cluster since this parameter is the determinant in differentiation for the construction of the clusters as well as modeling 3D protein structures for proteins not resolved via any crystallographic methodology. The evolution of the transmembrane domains of HAK proteins has been shaped by natural selection processes and environmental pressures over millions of years. These domains, which are part of the protein structure responsible for K^+^ transport across the cell membrane, have undergone adaptive changes to optimize K^+^ uptake and efficiency under various abiotic and biotic stress conditions. Recent research [48] has revealed the genetic diversity of the transmembrane domains of HAK proteins in plant species, highlighting the presence of allelic variants associated with different K^+^ transport capacities and adaptation to adverse soil conditions, such as salinity and drought. The findings from these studies suggest that the evolution of the transmembrane domains of HAK proteins is influenced by natural selection in response to environmental variability over geological time. Some authors [38] provide compelling evidence for positive selection in the transmembrane domains of HAK proteins in halophyte plant species, which exhibit increased salinity tolerance compared with non-halophyte plants. Their study suggests that the evolution of the transmembrane domains of HAK proteins is driven by selective pressure exerted by saline soil conditions, which favors the accumulation of genetic variants that improve K^+^ uptake and retention in plant cells. It has been reported that germinating seeds cannot exclude Na^+^ or Cl^−^ or accumulate these elements in vacuoles as older seedlings can [15]. The expression of the *PaHAK2* gene in response to salinity at 60 mM NaCl was very different in the two avocado varieties during imbibition. The americana variety showed a drastic increase early in its imbibition time. This suggests the participation of *PaHAK2* in the osmotic adjustment occurring in the embryo in this period before root protrusion. In drymifolia, there was a substantial increase later in the imbibition period. These data suggest that *PaHAK2* participates in the embryo development process under saline stress. In drymifolia, *PaHAK2* gene expression at 60 Mm was always lower than in the control, suggesting that NaCl could be inhibiting the expression of *PaHAK2.* This reduced gene expression would result in an inability of drymifolia to undergo early osmotic adjustment, as can be observed in the americana variety. This difference in gene expression could explain why we observed greater seedling vigor in the americana variety. Our data suggest that the *PaHAK2* gene participates in osmotic adjustment during the seed imbibition period. After the imbibition period and during seed germination, the expression of *PaHAK2* decreased in both varieties to a minimum both under saline and control conditions until day 10, when there was an increase in expression in both varieties under the saline condition, which was possibly related to a new osmotic adjustment in the seedlings. It is worth noting that during seed germination, the expression of *PaHAK2* was higher in drymifolia than in americana. Such enhanced gene expression suggests that *PaHAK2* could participate in the seed developmental processes of drymifolia, while in americana, *PaHAK2*’s primary role is in osmotic adjustment. *PaHAK2* may favor plant establishment under saline conditions in the drymifolia and americana avocado varieties. Given that *PaHAK2* is an abundantly organ-specific expressed gene in immature avocado seeds, this gene likely has a role in seed development and the response to salt stress. Further studies are needed to verify the functionality of this HAK/KUP/KT avocado transporter. This gene would be useful for the selection of avocado salinity-tolerant materials.

## 5. Conclusions

The americana avocado variety was more tolerant to salinity during germination than the drymifolia variety. Although both varieties achieved similar germination rates, drymifolia showed a slower germination speed under saline stress. The *PaHAK2* gene participated in the tolerance to salinity and was regulated differently in both varieties during the germination process. Our data suggest that *PaHAK2* has an important role in seed germination and seedling establishment under salinity stress in avocado.

## Data Availability

Data are contained in this article.

## Supplementary Materials

The following supporting information can be downloaded at: https://www.mdpi.com/article/10.3390/life14121680/s1. Table S1. Germination responses in quantity and time of the americana variety at different NaCl concentrations. Table S2. Germination responses in quantity and time of the drymifolia variety at different concentrations of NaCl.

## References

[1] Hasegawa P.M. (2013). Sodium (Na^+^) homeostasis and salt tolerance of plants. Environ. Exp. Bot..

[2] Van Zelm E., Zhang Y., Testerink C. (2020). Salt tolerance mechanisms of plants. Ann. Rev. Plant Biol..

[3] Mickelbart M.V., Melser S., Arpaia M.L. (2007). Salinity-induced changes in ion concentrations of ‘Hass’ avocado trees on three rootstocks. J. Plant Nutr..

[4] González-Rosas H., Salazar-García S., Ramírez-Reyes G., Rodríguez-Ontiveros J.L., Ramos-Villaseñor A.C. (2003). Preliminary results on in vitro selection for tolerant to chloride excess in avocado. Rev. Chapingo Ser. Hort..

[5] Pliego-Alfaro F., Palomo-Ríos E., Mercado J.A., Pliego C., Barceló-Muñoz A., López-Gómez R., Hormaza J.I., Litz R.E., Litz R.E., Pliego-Alfaro F., Hornaza J.I. (2020). Persea americana Avocado. Biotechnology of Fruit and Nut Crops.

[6] Álvarez-Acosta C., Marrero-Dominguez A., Gallo-Llobet L., Gonzalez-Rodriguez A.M. (2018). Physiological response of selected avocados (*Persea americana*) subjected to NaCl and NaHCO_3_ stress. Sci. Hort..

[7] Castro V.M., Iturrieta E.R., Fassio O.C. (2009). Rootstock Effect on the Tolerance of cv. Hass Avocado Plants to NaCl Stress. Chil. J. Agric. Res..

[8] Lazare S., Yasuor H., Yermiyahu U., Kuhalskaya A., Brotman Y., Ben-Gal A., Dag A. (2021). It takes two: Reciprocal scion-rootstock relationships enable salt tolerance in ’Hass’ avocado. Plant Sci..

[9] Acosta-Rangel A.M., Li R., Celis N., Suarez D.L., Santiago L.S., Arpaia M.L., Mauk P.A. (2019). The physiological response of ‘Hass’ avocado to salinity as influenced by rootstock. Sci. Hort..

[10] Ramírez-Guerrero L.G., González-Rosas H., Calderón-Zavala G., Velázquez-Mendoza J., Cetina-Alcalá V.M., Castillo-González A.M., Delgado-Alvarado A. (2010). Efecto de NaCl y CaCl_2_ en el desarrollo de ejes embrionarios de *Persea americana* Mill. criollo y ’Hass’ cultivados in vitro. Rev. Chapingo Ser. Hort..

[11] Lascano H.R., Antonelli G.E., Luna C.M., Melchiorre M.N., Gómez L.D., Racca R.W., Trippi V.S., Casano L.M. (2001). Antioxidant system response of different wheat cultivars under drought field and in vitro studies. Aust. J. Plant Phys..

[12] Meza N., Arizaleta M., Bautista D. (2007). Efecto de la Salinidad en la Germinación y Emergencia de Semillas de Parchita (*Passiflora edulis* f. *flavicarpa*). Rev. Fac. Agron..

[13] Rajabi Dehnavi A., Zahedi M., Ludwiczak A., Cardenas Perez S., Piernik A. (2020). Effect of salinity on seed germination and seedling development of Sorghum (*Sorghum bicolor* (L.) *Moench*) *genotypes*. Agronomy.

[14] Berkessa A.J. (2020). Salinity and Avocado Production, A Review. Int. J. For. Hort..

[15] Grieve C.M., Grattan S.R., Maas E.V., Wallender W.W., Tanji K.K. (2012). Plant salt tolerance. Agricultural Salinity Assessment and Management.

[16] He Y., Yang B., He Y., Zhan C., Cheng Y., Zhang J., Zhang H., Cheng J., Wang Z. (2019). A quantitative trait locus, qSE3, promotes seed germination and seedling establishment under salinity stress in rice. Plant J..

[17] Pliego-Alfaro F., Barcelo-Muñoz A., López-Gómez R., Ibarra-Laclette E., Herrera-Estrella L., Palomo-Ríos E., Mercado J.A., Litz R.E., Schaffer B., Wolstenholme B.N., Whiley. A.W. (2013). Biotechnology. The Avocado Botany, Production and Uses.

[18] Ibarra-Laclette E., Méndez-Bravo A., Pérez-Torres C.A., Albert V.A., Mockaitis K., Kilaru A., López-Gómez R., Cervantes-Luevano J.I., Herrera-Estrella L. (2015). Deep sequencing of the Mexican avocado transcriptome, an ancient angiosperm with a high content of fatty acids. BMC Genom..

[19] López-Gómez R., Suáres-Rodríguez L.M., Ibarra-Laclette E., Guzmán-Rodríguez J.J., López-Meza J.E., Ochoa-Zarzosa A., Salgado-Garciglia R., Rodríguez-Zapata L.C., Jimenéz-Moraila B., Herrera-Estrella L. (2016). Transcriptome (ESTs) of native Mexican avocado fruit is dominated by stress and innate immunity genes. Acta Hort..

[20] Suárez-Rodríguez L.M., Sánchez-Albarrán F., León-Corona H., López-Gómez R., Jimenez-Lopez J.C. (2017). Transcriptome (ESTs) of Avocado “native” mexicano early seed development shows abundance of regulatory, antioxidant and defense genes. Advances in Seed Biology.

[21] Sánchez-González E.I., Hernández-Delgado S., Aguirre-Arzola V.E., Torres-Castillo J.A., Gutiérrez-Díez A. (2018). Etiquetas de secuencias expresadas diferenciales de frutos de aguacate raza mexicana (*Persea americana* Mill. var. *drymifolia*). Rev. Bras. Frutic..

[22] Vergara-Pulgar C., Rothkegel K., González-Agüero M., Pedreschi R., Campos-Vargas R., Defilippi B.G., Meneses C. (2019). De novo assembly of *Persea americana* cv. ’Hass’ transcriptome during fruit development. BMC Gen..

[23] Dreyer I. (2014). Potassium (K^+^) in plants. J. Plant Phys..

[24] Monder H., Maillard M., Chérel I., Zimmermann S.D., Paris N., Cuéllar T., Gaillard I. (2021). Adjustment of K^+^ Fluxes and Grapevine Defense in the Face of Climate Change. Int. J. Mol. Sci..

[25] Kronzucker H.J., Coskun D., Schulze L.M., Wong J.R., Britto D.T. (2013). Sodium as nutrient and toxicant. Plant Soil.

[26] Munns R., James R.A., Gilliham M., Flowers T.J., Colmer T.D. (2016). Tissue tolerance: An essential but elusive trait for salt-tolerant crops. Funct. Plant Biol..

[27] Shen Y., Shen L., Shen Z., Jing W., Ge H., Zhao J., Zhang W. (2015). The potassium transporter *OsHAK21* functions in the maintenance of ion homeostasis and tolerance to salt stress in rice. Plant Cell Environ..

[28] Nieves-Cordones M., Ródenas R., Chavanieu A., Rivero R.M., Martinez V., Gaillard I., Rubio F. (2016). Uneven HAK/KUP/KT Protein Diversity Among Angiosperms: Species Distribution and Perspectives. Front. Plant Sci..

[29] Wang Z., Wang J., Bao Y., Wu Y., Zhang H. (2011). Quantitative trait loci controlling rice seed germination under salt stress. Euphytica.

[30] Wang Y., Chen L., Song G., Huang T., Wu G., Tan J., Wang P., Cheng Q., Li C., Zhong Q. (2022). Localization of salt-tolerant QTL in rice germination stage under different salinity concentrations. Euphytica.

[31] Zhao Y., Wang L., Zhao P., Liu Z., Guo S., Li Y., Liu H. (2022). Genome-wide identification, characterization, and expression analysis of HAK genes and decoding their role in responding to potassium deficiency and abiotic stress in *Medicago truncatula*. PeerJ.

[32] Wang Z., Hong Y., Zhu G., Li Y., Niu Q., Yao J., Hua K., Bai J., Zhu Y., Shi H. (2020). Loss of salt tolerance during tomato domestication conferred by variation in a Na^+^/K^+^ transporter. EMBO J..

[33] Elekou E.A.C., Perea-Arango I., Suarez-Rodriguez L.M., López-Gómez R., Jimenez-Lopez J.C. (2022). In Vitro seed germination and seedling development of two avocado varieties. Seed Biology Updates.

[34] Murashige T., Skoog F.A. (1962). Revised Medium for Rapid Growth and Bio Assays with Tobacco Tissue Cultures. Plant Phys..

[35] González-Zertuche L., Orozco-Segovia A. (1996). Methods for seed germination data analysis. An example: Manfreda brachystachya. Bot. Sci..

[36] Barbier F.F., Chabikwa T.G., Ahsan M.U., Cook S.E., Powell R., Tanurdzic M., Beveridge C.A. (2019). A phenol/chloroform-free method to extract nucleic acids from recalcitrant, woody tropical species for gene expression and sequencing. Plant Met..

[37] StatSoft Inc (2011). Statistica. System Reference.

[38] Rehman H.M., Nawaz M.A., Shah Z.H., Daur I., Khatoon S., Yang S.H., Chung G. (2017). In-Depth Genomic and Transcriptomic Analysis of Five K^+^ Transporter Gene Families in Soybean Confirm Their Differential Expression for Nodulation. Front. Plant Sci..

[39] Hasegawa P.M., Bressan R.A., Zhu J.K., Bohnert H.J. (2000). Plant cellular and molecular responses to high salinity. Ann. Rev. Plant Phys. Plant Mol. Biol..

[40] Ibrahim E.A. (2016). Seed priming to alleviate salinity stress in germinating seeds. J. Plant Physiol..

[41] Bernstein N., Ioffe M., Zilberstaine M. (2001). Salt-stress effects on avocado rootstock growth. I. Establishing criteria for determination of shoot growth sensitivity to the stress. Plant Soil.

[42] Bayuelo-Jiménez J.S., Craig R., Lynch J.P. (2002). Salinity tolerance of phaseolus species during germination and early seedling growth. Crop Sci..

[43] Sholi Nasser J.Y. (2012). Effect of Salt Stress on Seed Germination, Plant Growth, Photosynthesis and Ion Accumulation of four Tomato Cultivars. Am. J. Plant Phys..

[44] Nasri N., Maatallah S., Kaddour R., Lachaal M. (2016). Effect of salinity on *Arabidopsis thaliana* seed germination and acid phosphatase activity. Arch. Biol. Sci..

[45] Murillo A.B., Troyo D.E., López C.A., Jones H.G., Ayala C.F., Tinoco O.C.L. (2001). Salt tolerance of cowpea genotypes in the emergence stage. Aust. Prod. Sci..

[46] Jamil M., Lee D.B., Jung K.Y., Muhammad A., Lee S.C.H., Rha E.S. (2006). Effect of salt (NaCl) stress on germination and early seedling growth of four vegetables species. JCEA.

[47] Munns R., Tester M. (2008). Mechanisms of salinity tolerance. Ann. Rev. Plant Biol..

[48] Wang Y., Zhang Y., Wei Y., Meng J., Zhong C., Fan C. (2023). Characterization of HAK protein family in *Casuarina equisetifolia* and the positive regulatory role of *CeqHAK6* and *CeqHAK11* genes in response to salt tolerance. Front. Plant Sci..

