# Supplementary material for: Characterization of the PaHAK Gene and Its Expression During the In Vitro Seed Germination of Two Botanical Avocado Varieties Under Saline Stress
Source: Life (Basel). 2024 Dec 18;14(12):1680. doi: 10.3390/life14121680 (PMC11677014; doi:10.3390/life14121680)
Supplement: Supplementary file 1 [file life-14-01680-s001.zip › Table S2 Germination drymifolia.pdf]

Table S2. Germination responses in quantity and time of the drymifolia variety at different concentrations of NaCl

Drymifolia variety								
**NaCl (mM)**	**T25_CG**	**T25_TG**	**T50_CG**	**T50_TG**	**T75_CG**	**T75_TG**	**Seedlings %**	**GS**
**0**	11.5 ± 0.50 **a**	3.83 ± 0.08 **a**	19.67 ± 0.33	4.67 ± 0.17 **a**	19.67 ± 0.33	5.75 ± 0.25 **a**	98.33 ± 1.67	2.58 ± 0.14 **a**
**15**	7.5 ± 0.50 **b**	4.17 ± 0.30 **a**	18.67 ± 0.67	5.33 ± 0.33 **ab**	19.67 ± 0.33	6.75 ± 0.38 **ab**	98.33 ± 1.67	2.21 ± 0.17**ab**
**30**	5.83 ± 0.67 **b**	4.58 ± 0.30 **a**	17.33 ± 0.88	6 ± 0.00 **b**	19.33 ± 0.67	7.08 ± 0.58 **ab**	96.67 ± 3.33	1.93 ± 0.07 **b**
**60**	2.50 ± 0.50 **c**	6 ± 0.00 **b**	15.33 ± 1.76	7 ± 0.00 **c**	18.67 ± 0.33	8 ± .58 **b**	93.33 ± 1.67	1.94 ± 0.09 **b**

Comparison between treatments (0, 15, 30, and 60 mM NaCl); T25, T50, T75: Time corresponding to 25%, 50%, and 75% of the germinated seeds (Days) ; CG: Cumulative germination; TG: Average germination time; GS: Germination speed. Different letters indicate a significant difference from the control plants ($p < 0.05$)
