# Supplementary material for: Characterization of the PaHAK Gene and Its Expression During the In Vitro Seed Germination of Two Botanical Avocado Varieties Under Saline Stress
Source: Life (Basel). 2024 Dec 18;14(12):1680. doi: 10.3390/life14121680 (PMC11677014; doi:10.3390/life14121680)
Supplement: Supplementary file 1 [file life-14-01680-s001.zip › Table S1 Germination americana.pdf]

Table S1. Germination responses in quantity and time of the americana variety at different NaCl concentrations.

Americana variety								
**NaCl (mM)**	**T25_CG**	**T25_TG**	**T50_CG**	**T50_TG**	**T75_CG**	**T75_TG**	**Seedlings %**	**GS**
**0**	13.83 ± 0.83 **a**	3.58 ± 0.30 **a**	20 ± 0.00	4.33 ± 0.17 **a**	20 ± 0.00	5 ± 0.00 **a**	100 ± 0.00	3.33 ± 0.00 **a**
**15**	10.33 ± 0.83 **b**	4 ± 0.00 **ab**	19.67 ± 0.33	5 ± 0.00 **ab**	19.67 ± 0.33	5.75 ± 0.25 **ab**	98.33 ± 1.67	2.96 ± 0.10 **b**
**30**	8.5 ± 0.58 **b**	4.5 ± 0.25 **bc**	19.33 ± 0.33	5.33 ± 0.33 **ab**	19.33 ± 0.33	6.00 ± 0.00 **b**	96.67 ± 1.67	2.76 ± 0.05 **b**
**60**	7.5 ± 0.29 **b**	5 ± 0.00 **c**	19.67 ± 0.33	5.17 ± 0.17 **b**	19.67 ± 0.33	6.33 ± 0.**33** b	98.33 ± 1.67	2.81 ± 0.05 **b**

Comparison between treatments (0, 15, 30 and 60 mM NaCl);; T25, T50, T75: Time corresponding to 25%, 50%, and 75% of the germinated seeds (Days) ; CG: Cumulative germination; TG: Average germination time; GS: Germination speed. Different letters indicate a significant difference from the control plants ($p < 0.05$).
